# Mushroom-derived polysaccharides as broad-spectrum antimicrobial agents: mechanisms, structural modifications, and therapeutic potential

**DOI:** 10.3389/fmicb.2026.1833457

**Published:** 2026-06-11

**Authors:** Meifang Peng, Xinrui Kan, Wenli Huang

**Affiliations:** 1 Biotechnology and Nuclear Technology Research Institute, Sichuan Academy of Agricultural Sciences, Chengdu, China; 2 College of Food and Biological Engineering, Chengdu University, Chengdu, Sichuan, China

**Keywords:** antimicrobial activity, immunomodulation, mushroom polysaccharides, structural modification, structure–activity relationship, *β*-glucan

## Abstract

Antibiotic resistance poses one of the most pressing threats to global public health, underscoring the urgent need for novel antimicrobial agents. Mushroom-derived polysaccharides have attracted considerable attention due to their structural diversity, low toxicity and various pharmacological activities. This review provides a comprehensive overview of the antiviral, antibacterial, and antifungal activities of polysaccharides isolated from edible and medicinal mushrooms against a range of pathogens, including multidrug-resistant species, with particular emphasis on the structural features that govern their efficacy. The mechanisms underlying these activities are examined, encompassing host immunomodulation, inhibition of pathogen attachment and entry, enzymatic inhibition of microbial replication and direct disruption of microbial cell integrity. Strategies to enhance antimicrobial potency are also discussed, covering chemical derivatization (e.g., sulfation, deacetylation), nanocomposite formulation and combination with conventional antimicrobials. Finally, the challenges of clinical translation including limited oral bioavailability, structural heterogeneity across preparations, and the lack of randomized clinical trial data in infectious disease indications, are critically evaluated. Addressing these gaps through standardization of production protocols, integrative structure–activity characterization and well-designed clinical trials will help advance the application of mushroom polysaccharides in medicine, food preservation, and agriculture.

## 1 Introduction

The global burden of infectious diseases remains substantial, and the accelerating emergence of multidrug-resistant pathogens has critically undermined the effectiveness of existing antimicrobial therapies across clinical, agricultural, and veterinary settings (Brussow, 2024). There is an urgent need to discover and develop novel antimicrobial agents that can complement or serve as alternatives to conventional antibiotics. Natural products have historically played a central role in drug discovery, particularly in the search for new antimicrobial compounds. It is estimated that more than 80% of currently available antibiotics are derived from natural sources, including plants, lichens, actinomycetes, and fungi (Elmaidomy et al., 2022). Among these, fungi are especially noteworthy for their biosynthetic capabilities, producing structurally diverse metabolites with potent antimicrobial activities (Mishra and Tiwari, 2011). Classic antibiotics such as penicillin and cephalosporins were originally isolated from fungal species, highlighting their historical and continuing importance in the development of antimicrobial agents (Demain and Sanchez, 2009).

Mushrooms, a group of macroscopic fungi that produce fruiting bodies, have long been valued not only as nutritional foods but also as traditional medicinal resources in Asia. Well-known species such as *Ganoderma lucidum*, *Lentinus edodes* (Shiitake) and *Grifola frondosa* (Maitake) have been used for centuries owing to their immunomodulatory, hepatoprotective, and anti-infective effects (Hamad et al., 2022; Chowdhury et al., 2015; Khadhri et al., 2017). Over the past few decades, these traditional claims have been increasingly substantiated by modern pharmacological studies, many of which have provided experimental evidence for their bioactivity. Mushrooms are now recognized as rich sources of bioactive metabolites, among which polysaccharides have attracted particular attention due to their structural complexity and diverse biological functions. These polysaccharides are typically high molecular weight carbohydrates composed of repeating monosaccharide units, often linked via *β*-glucan backbones with various branching patterns. They exhibit variable pharmacological properties, including immunomodulatory, antitumor, antioxidant, hypoglycemic, and notably antimicrobial activities (Maity et al., 2021; Friedman, 2016). Structurally, mushroom polysaccharides can be classified into *β*-glucans, heteropolysaccharides, glycoprotein complexes, and mannose-rich polymers, each group associated with distinct biological mechanisms (Wasser, 2011).

Recent studies have increasingly highlighted the antimicrobial potential of mushroom polysaccharides, demonstrating inhibitory effects against bacterial, fungal, and viral pathogens (Alves et al., 2012; Clericuzio et al., 2021). Antibacterial activity has been reported against both Gram-positive and Gram-negative species, including *Staphylococcus aureus* (particularly methicillin-resistant strains, MRSA) and *Escherichia coli*. Antifungal activities have been observed against opportunistic pathogens such as *Candida albicans* and *Aspergillus fumigatus*, with proposed mechanisms involving inhibition of mycelial growth, disruption of cell membrane integrity, and suppression of biofilm formation, which is a key factor contributing to microbial virulence and drug resistance. Although the antiviral properties of mushroom polysaccharides have been extensively reviewed (Guo et al., 2021; Teplyakova and Kosogova, 2016; He et al., 2020), a comprehensive synthesis of their antimicrobial activities across different pathogen types remains lacking. This review aims to provide a comprehensive and up-to-date overview of the antimicrobial potential of mushroom-derived polysaccharides, with particular emphasis on their structural features, mechanisms of action, structure–activity relationships, and prospective applications as novel antimicrobial agents.

## 2 Sources, extraction, and structural characteristics of mushroom-derived polysaccharides

### 2.1 Representative antimicrobial mushroom species

Mushrooms with antimicrobial activity span a wide range of taxonomic groups, reflecting the structural and functional diversity of their polysaccharides. Beyond their taxonomic interest, several genera are now recognized as sources of polysaccharides with documented activity against clinically important human pathogens, including those responsible for respiratory tract infections, chronic viral hepatitis, foodborne and nosocomial bacterial infections, herpesvirus reactivation disease, and emerging multidrug-resistant pathogens, providing a translational rationale for their continued investigation.

*Schizophyllum commune*, a ubiquitous wood-decomposing basidiomycete, represents one of the earliest and most intensively investigated species. Its extracellular *β*-glucan, schizophyllan, was among the first fungal polysaccharides reported to enhance host resistance against infection (Tabata et al., 1981; Komatsu et al., 1973). Its clinical relevance is anchored in both oncology and infectious-disease contexts: the purified preparation sizofiran (SPG) has been used in Japan as an immunoadjuvant in cervical cancer therapy, and clinical studies in chronic hepatitis B patients have demonstrated augmentation of HBV nucleocapsid-antigen–specific T- and B-cell responses. Although its direct bactericidal activity *in vitro* is relatively limited, schizophyllan has been demonstrated to prolong survival in experimental models of pulmonary tuberculosis and other bacterial and fungal infection (Matsumiya and Saito, 1977). Subsequent studies have confirmed its potential as an adjuvant in combination therapies for respiratory and mycobacterial infections (Komatsu et al., 1975). In addition, schizophyllan-based membranes elaborated by crosslinking of amine-grafted bacterial cellulose have effectively inhibited the growth of microorganisms (Hamedi et al., 2020). Chemically modified derivatives, oxidized schizophyllan (scleraldehyde) have also exhibited potential antibacterial activity, including against enteric pathogens implicated in gastrointestinal disease such as *Shigella sonnei*, *Salmonella typhi*, and *Vibrio cholerae* (Jayakumar et al., 2010).

*Ganoderma* species, particularly *G. lucidum*, occupy a central place in East Asian traditional medicine and have been extensively examined for their antimicrobial potential. Within infectious-disease contexts, *Ganoderma* polysaccharides have been most consistently evaluated against chronic viral hepatitis (HBV-related liver disease) and opportunistic bacterial pathogens of the skin and respiratory tract. Numerous studies have summarized the antimicrobial activity of extracts and bioactive compounds from *Ganoderma* spp. against human pathogenic microorganisms (Rai et al., 2015). However, findings concerning the specific contribution of polysaccharides remain inconsistent. While exopolysaccharides from culture filtrates and crude extracts from fruiting bodies have demonstrated moderate antibacterial effects (Robles-Hernández et al., 2021; Mahendran et al., 2013; Skalicka-Woźniak et al., 2012), polysaccharides isolated from mycelia often show limited direct antimicrobial activity (Wan-Mohtar et al., 2016). This discrepancy likely reflects compositional and methodological confounding rather than a fundamental absence of activity. Crude extracts and culture filtrates contain triterpenoids, phenolics, and glycoproteins that may contribute independently or synergistically to observed antibacterial effects, making it difficult to attribute activity specifically to polysaccharide fractions. In addition, mushroom polysaccharides frequently occur as polysaccharide–protein complexes or proteoglycans, and their biological activity is often dependent on these conjugated forms. Extensive purification procedures may remove associated proteins or disrupt higher-order conformations, potentially leading to reduced receptor recognition and diminished bioactivity. Structural differences between mycelial and fruiting-body polysaccharides in terms of molecular weight, branching degree, and glycosidic linkage patterns further limit cross-study comparability (Zhang et al., 2025). Other species such as *G. applanatum* (Osińska-Jaroszuk et al., 2014) and *G. formosanum* (Wang et al., 2011) exhibit selective antibacterial effects, against clinically relevant Gram-positive pathogens including *Staphylococcus aureus* and the foodborne intracellular pathogen *Listeria monocytogenes*, whereas *G. neo-japonicum* displays notable antiviral properties against enteroviruses responsible for hand, foot, and mouth disease (HFMD), an important pediatric infection in Asia (Ang et al., 2021). Collectively, these findings suggest that *Ganoderma* polysaccharides exert more pronounced immunomodulatory and antiviral rather than direct antibacterial effects. This pattern does not preclude therapeutic relevance, however, as preparations with modest *in vitro* potency may still confer clinical benefit through host immune activation—a mode of action that standard antimicrobial assays are not designed to detect (Karunarathna et al., 2025).

Another important genus is *Pleurotus* (oyster mushrooms), one of the most broadly cultivated edible fungi for their nutritional value and therapeutic potential. The polysaccharide, pleuran from *P. ostreatus*, improved survival in murine *L. monocytogenes* and influenzae infection models relevant to invasive listeriosis and respiratory disease (Wolff et al., 2008). Several other species, including *P. ostreatus P. eryngii* (Li and Shah, 2014), *P. eous* (Gunasekaran et al., 2021), *P. abalonus* (Wang et al., 2011), *P. tuber-regium* (Zhang et al., 2003; Zhang et al., 2004) and *P. florida* (Sen et al., 2013), have been investigated for their polysaccharides with promising antimicrobial abilities. These polysaccharides, typically isolated from fruiting bodies, mycelia and sclerotia, have been examined for their immunomodulatory and antimicrobial abilities *via* both *in vitro* and *in vivo* models. The broad adaptability and high productivity of *Pleurotus* species further support their potential as candidates for large-scale production of antimicrobial polysaccharides.

*Lentinula edodes* (shiitake) is one of the most commercially important mushrooms worldwide. Its principal polysaccharide, lentinan, isolated from fruiting bodies, has been extensively characterized for its immunoregulatory and antimicrobial properties (Sharma et al., 2016; Wang et al., 2013). Lentinan has been clinically approved as an immunoadjuvant in advanced gastric cancer chemotherapy. Although its formal clinical indications to date have been oncological, infectious-disease applications have included adjuvant therapy for chronic hepatitis B and preclinical efficacy against antibiotic-resistant *K. pneumoniae* pulmonary sepsis. Polysaccharide fractions obtained from mycelia and cultivation substrates, such as KS-2 (Suzuki et al., 1979) and PL2 (Zhu et al., 2012), have demonstrated activities relevant to infection control and host defense enhancement. In addition, films embedded with *L. edodes* stalk polysaccharides (LEP) exhibit stronger antibacterial activity against foodborne spoilage and pathogenic organisms, while preventing browning and softening of fresh mushrooms (Guo et al., 2023).

*Agaricus brasiliensis* (syn. *Agaricus blazei Murill*) and related species also serve as valuable sources of bioactive polysaccharides. Polysaccharide fractions and their sulfated derivatives are well characterized for activity against herpesviruses underlying recurrent orolabial and genital lesions (Minari et al., 2011; Faccin et al., 2007; Ren et al., 2018). Moreover, chitin–glucan complexes from *A. bisporus* have shown notable antibacterial activity against a panel of nosocomial pathogens including *S. aureus*, *P. aeruginosa*, and *E. coli* (Singh and Dutta, 2017).

Beyond these major genera, other mushroom species, including *Armillariella tabescens* (Zhang et al., 2022), *Grifola frondosa* (Maitake) (Zhang et al., 2017), *Auricularia auricula*-*judae* (Cai et al., 2015), *Coriolus versicolor* (Shi et al., 2016; Shi et al., 2022), *Fomitiporia punctata* (Liu et al., 2017), *Polyporus umbellatus* (Guo et al., 2019), *Armillaria luteo-virens* (Kurskaya et al., 2022), and *Hericium erinaceus* (Wu et al., 2018), have also been identified as potential sources of antimicrobial polysaccharides. Collectively, these findings highlight the versatility of edible and medicinal mushrooms as renewable resources for the development of bioactive polysaccharides with pharmacological applications.

### 2.2 Production, extraction, and purification of bioactive polysaccharides

The production and recovery of mushroom-derived polysaccharides involve cultivation, extraction, and purification, each of which profoundly affects the yield, structural integrity, and biological activity (Yang et al., 2022). The biosynthesis of polysaccharides is highly sensitive to environmental and nutritional parameters. Factors such as substrate composition, carbon/nitrogen ratio, mineral supplementation, and the presence of elicitors influence not only the total yield but also the physicochemical characteristics of the products (Skalicka-Woźniak et al., 2012). For instance, zinc supplementation during the cultivation of *G. frondosa* enhances the accumulation of zinc-enriched intracellular polysaccharides with improved antibacterial and antioxidant properties (Zhang et al., 2017). Similarly, the addition of elicitors in submerged cultures of *Cordyceps militaris* significantly increases both polysaccharide yield and its antimicrobial potency (Ding et al., 2023). For *G. lucidum*, variations in medium composition have been shown to alter exopolysaccharide productivity, carbohydrate content and elemental composition, which correlate with changes in antibacterial efficacy (Mahendran et al., 2013). These observations highlight that cultivation conditions exert a direct influence on both the yield and bioactivity of mushroom polysaccharides.

Mushroom polysaccharides are commonly extracted using hot-water extraction followed by ethanol precipitation, a simple and efficient method for isolating water-soluble fractions. Extraction parameters, including temperature, duration, and solvent-to-solid ratio, must be carefully optimized to maximize yield while preserving structural features (Sharma et al., 2016; Cai et al., 2015). Alkaline extraction is employed to recover insoluble or cell wall-bound polysaccharides. However, the harsh conditions may cause partial degradation or structural alteration, leading to reduced biological activity. Alkaline extraction and certain deproteinization procedures such as trichloroacetic acid precipitation can further compromise structural integrity by disrupting triple-helix conformations important for receptor engagement (Zhang et al., 2003; Yehia, 2022; Karácsonyi and Kuniak, 1994). Ultrasonic-assisted extraction can significantly improve recovery efficiency. For example, ultrasound treatment increased polysaccharide yield from *L. edodes* by 1.62-fold compared to conventional extraction (Zhao et al., 2018). Furthermore, ultrasonic depolymerization of schizophyllan preserved its bioactivity despite a decrease in molecular weight (Tabata et al., 1981). Microwave-assisted extraction and enzymatic hydrolysis have been applied for efficient recovery of intracellular polysaccharides and bound polysaccharide complexes (Savin et al., 2020; Geng et al., 2024). In addition, emerging green technologies, such as pulsed electric field-assisted, aqueous two-phase extraction, offer environmentally friendly and potentially selective alternatives for extraction of bioactive polysaccharides (Leong et al., 2021). However, their application to antimicrobial polysaccharides remains unexplored and warrants further investigation.

Following extraction, purification is essential to remove proteins, polyphenols, pigments, and low molecular weight impurities that may interfere with biological assays or therapeutic evaluation. Deproteinization is commonly achieved using the Sevag method or trichloroacetic acid precipitation, while dialysis or ultrafiltration effectively eliminate free sugars and small metabolites (Gunasekaran et al., 2021; Zhao et al., 2016). For further refinement, ion-exchange chromatography (e.g., DEAE–cellulose) separates polysaccharides based on charge, while size-exclusion chromatography (e.g., Sephadex G-200, Superdex) enables molecular weight fractionation (Ren et al., 2018). These sequential purification steps yield structurally homogeneous fractions suitable for detailed characterization and reproducible evaluation of antimicrobial activity. Despite these methodological advances, the absence of standardized extraction and purification protocols remains a fundamental obstacle to reproducible research. Polysaccharides produced under the same name, such as ‘Lentinan’ or ‘GLP’, may differ substantially in molecular weight distribution, branching degree, glycosidic linkage composition and residual protein content, depending on the source material, extraction conditions, and purification steps employed (Zhang et al., 2025). Without consistent manufacturing procedures, the physicochemical and structural properties of the final product are difficult to control, and meaningful comparison of biological activities across studies becomes unreliable. Establishing standardized, reproducible production protocols is therefore a prerequisite for generating comparable and translatable activity data.

### 2.3 Chemical features and monosaccharide composition

The chemical structure and monosaccharide compositions of mushroom-derived polysaccharides are fundamental determinants of their antimicrobial efficacy and biological specificity. These macromolecules exhibit remarkable structural diversity, arising from variations in glycosidic linkage type, monosaccharide profiles, molecular weight, and conformational architecture.

Among the most extensively studied are *β*-glucans, particularly *β*-(1 → 3)-glucans with *β*-(1 → 6) branching, which form the structural backbone of lentinan from *L. edodes* (Ren et al., 2018), schizophyllan from *S. commune* (Tabata et al., 1981) and pleuran from *P. ostreatus* (Karácsonyi and Kuniak, 1994). These β-glucans share a conserved *β*-(1 → 3)-glucopyranosyl main chain, but differ in branching degree and spatial configuration, which influence solubility, chain flexibility, and supramolecular organization of polysaccharides. *β*-(1 → 4) or β-(1 → 2) linkages have also been found in antimicrobial polysaccharide from *C. versicolor* (Shi et al., 2022) and *A. brasiliensis* (Cardozo et al., 2013). Many β-glucans can form higher-order conformations such as triple helices stabilized by interchain hydrogen bonding and physical cross-linking (Han et al., 2020). This triple-helix conformation is particularly significant because it facilitates binding to pattern recognition receptors such as Dectin-1, thereby enhancing immune activation in infection control.

Heteropolysaccharides derived from mushrooms exhibit greater complexity, composed of multiple monosaccharides, commonly D-galactose, D-mannose, L-fucose, L-arabinose, and rhamnose in varying molar ratios. For example, an exopolysaccharide from *G. formosanum* consists predominantly of mannose, galactose and glucose, with minor amounts of arabinose and fucose, forming a highly branched mannogalactan structure that differs from the glucose-rich polysaccharides of *G. lucidum* (Wang et al., 2011). A novel heteropolysaccharide (GFP1) isolated from the mycelia of *G. frondosa*, composed of a (1 → 6)-*β*-D-glucan backbone with a single (1 → 3)-*α*-D-fucopyranosyl branch, demonstrated the ability to inhibit the replication of enterovirus 71 (EV71) (Zhao et al., 2016). Accumulating evidence suggests that both the diversity and molar ratios of constituent monosaccharides, particularly galactose and mannose, strongly modulate immunological and antimicrobial activity (Ren et al., 2018). Sulfation of heteropolysaccharides P3a was found to change the molar percentage of monosaccharide and enhance the antimicrobial activity, indicating that post-extraction processing can influence both structure and bioactivity (Gunasekaran et al., 2021).

The molecular weight (MW) of polysaccharides significantly influences their biological activities. Medium to high MW fractions (approximately 50–600 kDa) generally exhibit stronger bioactivities than low MW counterparts. Among three fractions isolated from *G. formosanum*, the 6–52 kDa fraction induced the highest tumor necrosis factor-*α* (TNF-α) production in macrophages, outperforming both smaller and much larger polysaccharide fractions (Wang et al., 2011). Similarly, high MW fractions (215.3 kDa) from *L.edodes* showed stronger anti-HBV effects than lower MW ones (39.8 kDa) (Zhao et al., 2018). High MW glucans are often more efficiently recognized by immune cell receptors, thereby enhancing macrophage activation and cytokine release. However, extremely large molecules (>2000 kDa) may suffer from poor solubility and limited bioavailability, while very low MW glucans (<10 kDa), which lack extensive side chains, tend to show reduced bioactivity and rely on chemical modification or administration with immune co-stimulators to improve cell penetration and receptor interaction (Gunasekaran et al., 2021; Han et al., 2020). Therefore, maintaining an optimal molecular weight range is critical for ensuring both structural stability and efficient receptor binding, ultimately contributing to enhanced antimicrobial efficacy.

Structure–activity relationships in mushroom polysaccharides are complex and cannot be adequately captured by single parameter analyses. A systematic analysis of mushroom polysaccharides confirmed that antimicrobial and immunomodulatory bioactivities are governed by the simultaneous interplay of molecular weight, linkage pattern, branching degree, and higher-order conformation (Zhang et al., 2025). Monosaccharide composition establishes the chemical identity of the polysaccharide backbone and directly influences solubility, charge distribution, and interaction with host receptors. Glucose-based homopolysaccharides such as *β*-glucans exhibit strong immunomodulatory and antiviral activities, whereas heteropolysaccharides containing mannose, galactose, fucose, or arabinose residues may engage distinct receptor subtypes and display different activity profiles. Molecular weight exerts a profound influence on receptor engagement. Medium MW fractions interact most effectively with pattern-recognition receptors such as Dectin-1 and TLR2/TLR4, whereas excessively high MW restricts solubility and membrane accessibility, and low-weight fragments often lack the spatial architecture required for productive receptor binding. Glycosidic linkage configuration is equally critical. *β*-(1 → 3)-linked backbones with β-(1 → 6) branches adopt triple-helix conformations that are recognized by innate immune receptors and are associated with potent immunostimulatory activity, whereas *α*-linked configurations generally confer weaker biological responses. The degree and pattern of branching further modulate activity independently of backbone linkage, as highly branched structures expose more non-reducing termini that serve as ligand contact points. However, these parameters are not independent. For example, sulfation simultaneously increases charge density and disrupts chain conformation, altering receptor affinity in ways that cannot be predicted from degree of substitution alone, while depolymerization reduces molecular weight while unavoidably modifying branching architecture and conformation. The consequence is that SAR conclusions derived from one polysaccharide source rarely transfer to another without structural validation. Advancing structure-guided development of mushroom polysaccharide derivatives will require integrative multi-parameter analytical frameworks, combining monosaccharide composition, linkage analysis, molecular weight profiling and NMR-based conformational characterization, applied systematically across well-defined polysaccharide series.

In addition to single component polysaccharides, several polysaccharide complexes also contribute to the antimicrobial potential of mushrooms. The polysaccharide-peptide complex KS-2 from *L. edodes,* composed primarily of *α*-linked mannose with trace peptides, enhanced survival in influenza-infected mice by interferon production (Fujii et al., 1978). Similarly, the polysaccharopeptide (PSP) from *C. versicolor* exhibited inhibitory effects on HIV-1 replication (Collins and Ng, 1997). Yamamoto *et al.* demonstrated the antiherpetic activity of a *β*(1 → 6) and *α*(1 → 4) glucan–protein (PLS) and its sulfated derivative (SPLS) derived from *A. brasiliensis* (Yamamoto et al., 2013). Chitin–glucan complexes (ChGC) from *A. bisporus* show superior antibacterial activity compared with native chitin (Singh and Dutta, 2017). Furthermore, both the water-soluble melanin-glucan complex (MGC: 80% melanins and 20% beta-glucans) and the insoluble chitin–glucan–melanin complex (ChGMC: 70% chitin, 20% beta-glucans and 10% melanins) from *Fomes fomentarius* exhibit antibacterial, antifungal, and antiviral activities *in vitro* and *in vivo*, outperforming conventional drugs (Seniuk et al., 2011). These naturally derived hybrid polysaccharide complexes represent a distinct structural category that bridges compositional diversity and multifunctional bioactivity.

### 2.4 Structural modification and derivatization

Structural modification is a powerful approach to enhancing the antimicrobial potential of mushroom polysaccharides by improving their physicochemical characteristics such as solubility, membrane affinity, and molecular stability (Liu et al., 2023). These derivatization strategies often aim to optimize interactions with microbial cell membrane or immune receptors by altering molecular charge, hydrophobicity, or conformation of the parent polysaccharides (Han et al., 2008).

Sulfation is the most extensively studied chemical modifications for enhancing the biological activity of fungal polysaccharides. The introduction of sulfate groups significantly increases the negative charge density and hydrophilicity of the polysaccharide, thereby improving water solubility, promoting chain expansion and facilitating interactions with positively charged regions on microbial surfaces and viral envelope proteins (Ji et al., 2013; Ferreira et al., 2015). Sulfated *β*-glucans from *G. lucidum* (GS) exhibited dose-dependent inhibition against a broad panel of bacteria, including MRSA, whereas the native glucan showed no such activity (Wan-Mohtar et al., 2016). Sulfated derivatives of polysaccharide from *P. tuber-regium* (S-TM8) and lentinan (sLNT) potently blocked virus-host cell interactions by binding to viral surface glycoproteins (Zhang et al., 2003; Wang et al., 2013), and sulfation of acidic heteropolysaccharides P3a from *P. eous* consistently enhanced antibacterial efficacy relative to the native fraction (Gunasekaran et al., 2021). Across *P. eryngii*, *A. brasiliensis*, and *A. auricula*, sulfated derivatives have shown higher antiviral, antibacterial, antioxidant and anticancer activities compared to their native counterparts (Li and Shah, 2014; Zhang et al., 2003; Zhang et al., 2004; Yamamoto et al., 2013; Cardozo et al., 2014; Cardozo et al., 2011; Zhao et al., 2021). Notably, bioactivity is strongly influenced by the degree of substitution (DS) and site-specific substitution of sulfate groups. Optimal activity is typically observed at a DS of 1.0–1.5, which provides a balance between solubility, flexibility, and biological function. Substitutions generally occur at the C-6, C-2, or C-4 positions of sugar residues, depending on the sulfating reagent and substrate accessibility. Sulfation at the C-6 hydroxyl of galactose or glucose residues exerts the greatest effect on chain conformation and bioactivity (Li and Shah, 2014; Zhang et al., 2003; Zhang et al., 2004). Despite these pharmacological gains, several limitations constrain the translational relevance of sulfation derivatives. Most sulfation reactions employ chlorosulfonic acid or sulfur trioxide–pyridine complexes under conditions that are difficult to control precisely, resulting in heterogeneous products with variable degrees and patterns of substitution (Nguyen et al., 2012). This chemical heterogeneity complicates dose–response interpretation and undermines inter-study reproducibility. More critically, cytotoxicity of highly sulfated derivatives, particularly those with DS > 1.5, has not been systematically evaluated in mammalian cell models. Highly sulfated polysaccharides can exhibit anticoagulant activity analogous to heparin, a safety liability that warrants explicit investigation but has received insufficient attention in the literature, limiting clinical translation as therapeutic candidates. Accordingly, the clinical translation of sulfated polysaccharide derivatives requires not only precise control over sulfation processes with rigorous characterization of the degree and pattern of substitution, but also comprehensive evaluation of safety and efficacy, including cytotoxicity, anticoagulant activity, and pharmacokinetic behavior under physiologically relevant conditions.

Chitin, a naturally abundant amino polysaccharide in fungal cell walls, can be deacetylated chemically or enzymatically to produce chitosan, a well-studied derivative with improved solubility, bioavailability, and functional activity. The degree of N-deacetylation (DD) and MW are key determinants influencing its bioactivity. Chitosan with higher DD values carries more positive charges at physiological pH, facilitating stronger interactions with negatively charged bacterial membranes. However, the relationship between DD, MW, and bioactivity is not independent but context-dependent, varying with both the source and processing method. Chitosan derived from *L. edodes* stipes (DD: 75–85%; MW: 382–402 kDa) exhibits stronger antibacterial activity than crustacean derived chitosan, which has been attributed not only to a higher DD but also a more favorable MW distribution (Chien et al., 2016; Errington et al., 1993). Moreover, ultrasound-assisted deacetylation (USAD) of chitin from *G. lucidum* spore powder yielded chitosan with shorter molecular chains, which appears to enhance cellular penetration into bacterial cells, whereas thermochemical deacetylation (TCD) generates products with higher DD but reduced bioavailability (Zhu et al., 2018). Two chitosan preparations obtained from *G. lucidum* via enzymatic or alkaline pretreatment followed by deacetylation exhibited consistently high DD (>80%) and lower viscosity and MW compared to shrimp-derived counterparts (Savin et al., 2020). Notably, the enzymatically extracted chitosan displayed stronger radical scavenging activity and more favorable cytocompatibility, whereas the chemically extracted one exhibits more pronounced antibacterial effects. These observations indicate distinct bioactivity profiles that cannot be explained by DD or MW alone. A systematic bioactivity matrix analysis of chitosans with varying DD and MW has provided a comprehensive framework for rationalizing these structure–activity relationships (Eickelpasch et al., 2025), further supporting the importance of controlled deacetylation. These observations indicate that the origin, deacetylation method, and molecular architecture of chitosan critically determine its antimicrobial performance, and that findings from crustacean-derived chitosan cannot be assumed to transfer directly to fungal chitosan without structural validation.

**Figure 1:**
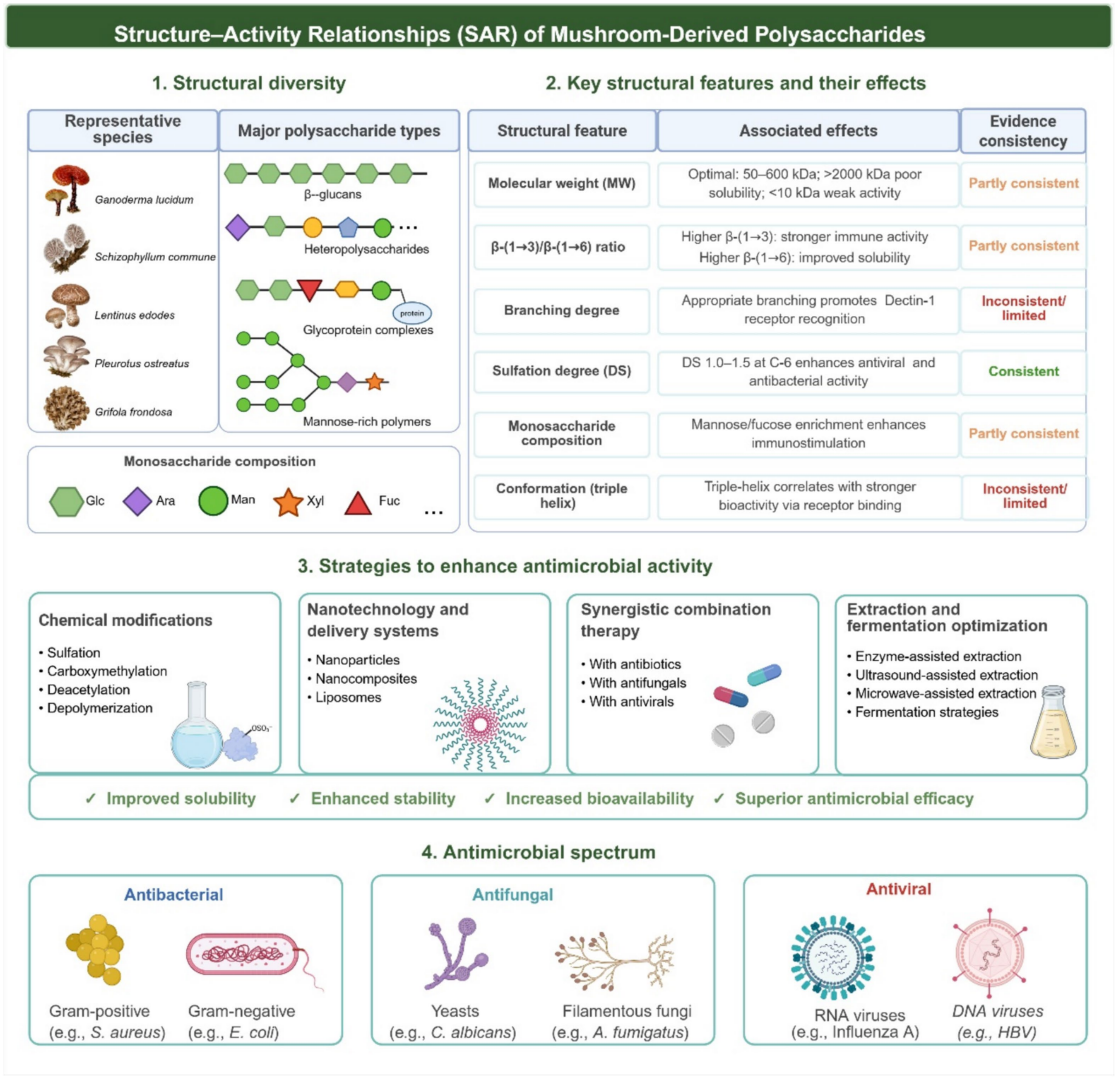
Structure–activity relationships (SAR) of mushroom-derived polysaccharides and strategies to enhance their antimicrobial activity. Antimicrobial activity is influenced by molecular weight, glycosidic linkage, branching degree, sulfation level, monosaccharide composition, and triple-helix conformation. Strategies to improve bioactivity include chemical modification, nanoparticle delivery, combination with conventional antimicrobials, and optimized extraction, resulting in broad-spectrum antibacterial, antifungal, and antiviral activities. Created with BioRender.com. A publication license has been obtained (Agreement number: BO29SUWQEJ), which permits publication under the Creative Commons Attribution License (CC-BY).

Incorporating mushroom polysaccharides into nanocomposites has emerged as a promising approach to improve their stability, delivery efficiency, and biological function. Polysaccharide-based nanocomposites often demonstrate enhanced antimicrobial properties due to improved surface reactivity, synergistic interactions, and better penetration across microbial cells membrane. *G. lucidum* polysaccharide nanoparticles (GLP-NPs) show stronger and longer-lasting antibacterial effects compared with GLP alone (Qin et al., 2018). Similarly, *Pleurotus* species have been effectively employed for the biogenic synthesis of polysaccharide-silver nanoparticles, which exhibit potent antimicrobial activity (Irshad et al., 2020). The silver nanoparticle *β*-glucan conjugates, synthesized from linear (1 → 6)-β-D-glucan from *P. florida* blue variant, effectively inhibited multidrug-resistant *Klebsiella pneumoniae* (Sen et al., 2013). Similarly, silver nanoparticles biosynthesized using polysaccharides extracted from *Phlebopus portentosus* have shown potent antibacterial activity alongside antidiabetic and anticancer effects (Li et al., 2024), expanding the functional scope of these nanocomposites. In addition, incorporation of iron (III) ions into *Flammulina velutipes* polysaccharides (FVP) yielded FVP-Fe nanocomplexes with strong inhibitory ability against multiple bacterial strains (Dong et al., 2017). Recent advances have further demonstrated that polysaccharide-based nanocarriers serve as effective delivery platforms for natural antimicrobial agents, enhancing their stability and bioavailability (Kotenkova et al., 2025). Nanofiber scaffolds fabricated from pomegranate peel polyphenols and *P. eryngii* polysaccharides effectively reduced *E. coli* colonization on meat and vegetable surfaces, suggesting antimicrobial potential for infection control and food safety (Cai et al., 2021). Lipid nanoparticles functionalized to target bacterial polysaccharides have demonstrated selective bactericidal activity against Gram-negative pathogens (Lai et al., 2023), representing a promising direction for the targeted delivery of mushroom polysaccharide-based therapeutics. Collectively, these studies indicate that nanocomposite engineering can substantially enhance the antimicrobial performance of mushroom polysaccharides. However, the heterogeneity of nanomaterials, the lack of standardized toxicity evaluation, and limited *in vivo* validation remain significant barriers to clinical translation (Figure 1).

## 3 Antimicrobial activities of mushroom polysaccharides

### 3.1 Activity against viruses

Mushroom polysaccharides have attracted extensive attention in recent years for their broad-spectrum antiviral properties (Maity et al., 2021). They exhibit inhibitory activities against both naked and enveloped viruses through multiple mechanisms, including direct viral inactivation, prevention of viral adsorption, and modulation of host immune responses.

#### 3.1.1 Influenza virus

Polysaccharides from various mushroom species demonstrate notable protective effects against influenza infection. Oral administration of *β*-glucans from *Trametes versicolor* reduced body weight loss, mortality and viral titers in influenza-infected mice, while alleviating the pathological lung damage. Comparable results were observed in chickens infected with H9N2 avian influenza virus, where β-glucans improved survival and decreased pulmonary viral loads (Shi et al., 2022). The polysaccharide-peptide complex KS-2 induced interferon production, improved survival, inhibited lung consolidation and reduced viral titers in mice infected with influenza A (H2N2), although it showed no direct virucidal or virustatic activity *in vitro* (Suzuki et al., 1979; Fujii et al., 1978). Moreover, water-soluble polysaccharides from *Hygrophorus* spp. (Vlasenko et al., 2021) and *A. luteo-virens* (Kurskaya et al., 2022) also demonstrated antiviral effects against influenza virus. Cell-based studies using MDCK cells provide direct evidence of viral replication inhibition. *In vivo* studies measure host survival, weight loss, and viral titers as composite endpoints that reflect both direct antiviral activity and polysaccharide-mediated immunostimulation.

#### 3.1.2 Herpesvirus

Polysaccharide from *A. brasiliensis* and its derivatives show remarkable antiherpetic activity against herpes simplex type 1 (HSV-1), herpes simplex type 2 (HSV-2) and bovine herpes type 1 (BoHV-1). The *β*-glucans and polysaccharide-peptide effectively inhibited BoHV-1 with the IC_50_ of 140 μg/mL and 160 μg/mL, respectively (Minari et al., 2011; Bruggemann et al., 2006). A polysaccharide fraction (F3), glucan–protein (PLS) and its sulfated derivative (SPLS) inhibited HSV-1 and BoHV-1 dose-dependently without virucidal or viral adsorption inhibition, suggesting interference with intracellular viral replication (Yamamoto et al., 2013). A notable pattern across herpesvirus studies is the marked contrast between native and sulfated polysaccharides. Most native polysaccharides are inactive, while sulfated derivatives achieve IC50 values in the microgram range. Sulfated derivatives FR-S and MI-S significantly suppressed HSV-1 and HSV-2 interactions with cell components and reduced viral adsorption and spread without direct virucidal effects, whereas native FR and MI did not present antiherpetic activity *in vitro* (Cardozo et al., 2013; Cardozo et al., 2014; Cardozo et al., 2011). MI-S also mitigated the HSV cutaneous and mucosal lesions *in vivo*, exhibiting a synergistic effect with acyclovir. Additionally, sulfated *β*-glucan TM8-S showed stronger inhibition of HSV-1 and HSV-2 with the IC_50_ of 0.125–2.5 μg/mL than acyclovir (Zhang et al., 2003; Zhang et al., 2004). This reliance on sulfation implies that electrostatic interactions with heparan sulfate proteoglycans on host cells are the primary mechanism of action, rather than an intrinsic effect of the polysaccharide itself. In addition, polysaccharide from *L. edodes* (LeP) inhibited BoHV-1 replication by up to 74.2% at 0.2 mg/mL when administered at the time of infection, without virucidal activity and inhibition of viral adsorption (Faccin et al., 2007; Rincao et al., 2012).

#### 3.1.3 Human immunodeficiency virus

Several mushroom-derived polysaccharides and their sulfated derivatives display promising activity against multiple steps of the HIV replication cycle. A purified *α*-D-glycan (LA, 120 kD) from *P. abalonus* inhibited HIV-1 reverse transcriptase with an IC_50_ of 8.7 × 10^−2^ μM (Wang et al., 2011). A novel polysaccharide fraction (G1, ~151 kDa) from *F. punctata* inhibited HIV-1 protease by 19.6% at 50 μg/mL, and suppressed integrase activity at 100–1000 μg/mL (Liu et al., 2017). The polysaccharopeptide PSP strongly inhibited recombinant HIV-1 reverse transcriptase, blocked interaction between HIV-l gp120 and immobilized CD4 receptor, and interfered with viral glycohydrolase activity essential for glycosylation (Collins and Ng, 1997). Chemically sulfated polysaccharides from *A. blazei* demonstrated potent anti-HIV activity with IC₅₀ values comparable to azidothymidine (Zhao et al., 2021). Furthermore, melanin–glucan complex MGC also showed strong anti-HIV-1 activity with a low toxicity, outperforming zidovudine (Retrovir) (Seniuk et al., 2011). The HIV inhibitory activity is primarily derived from cell-free enzyme assays rather than viral replication models, which can provide preliminary mechanistic data but cannot fully reflect the complex effects within a biological system. Cell-based confirmation of antiviral efficacy was generally lacking.

#### 3.1.4 Hepatitis virus

Mushroom polysaccharides demonstrate notable inhibitory effects against hepatitis B virus (HBV) through suppression of viral antigen secretion, inhibition of DNA replication, and enhancement of host immune responses. Polysaccharides LEP-1 and LEP-2 significantly reduced the secretion of hepatitis B surface antigen (HBsAg) and e antigen (HBeAg) in HepG2.2.15 cells (Zhao et al., 2018). Polysaccharides from *L. edodes* waste material (PLWM) also inhibited the secretion of HBsAg and HBeAg in HepAD38 and HepG2.2.15 cells, as well as improved the antiviral activity of lamivudine against HBV DNA replication (Jiao et al., 2018). Similar findings were observed in a water-soluble polysaccharide FVP1 (Zhang et al., 2018). Moreover, Sizofiran from *S. commune* (SPG) enhanced IFN-*γ* production and proliferation of peripheral blood mononuclear cells (PBMC) from chronic hepatitis B patients in response to HBV nucleocapsid antigens, and increased both anti-HBc and anti-HBe antibody production in antigen-stimulated cultures, highlighting its ability to modulate both cellular and humoral immune responses (Kakumu et al., 1991). Nevertheless, HepG2.2.15 cells, the predominant experimental model used in HBV polysaccharide studies, constitutively express HBV but do not replicate the immunological environment of the human liver, which limits the mechanistic relevance of *in vitro* findings. More physiologically relevant systems, such as HBV-infected HepaRG cells or patient-derived liver organoids, would significantly enhance the translational value.

#### 3.1.5 Enteroviruses

In addition to their activity against enveloped viruses, mushroom polysaccharides have been reported to inhibit several non-enveloped RNA viruses, particularly enteroviruses that cause neurological and systemic infections. A novel heteropolysaccharide (GFP1) from *G. frondosa* inhibited the replication of enterovirus 71 (EV71) by blocking viral VP1 protein expression and viral RNA synthesis (Zhao et al., 2016). LeP suppressed poliovirus type 1 (PV-1) replication by 63.8% at 0.2 mg/mL when administered at the onset of infection, whereas pre- or late-stage administration was less effective. This activity was accompanied by low virucidal activity and no inhibition of viral adsorption, indicating interference during early replication stages (Rincao et al., 2012). Similarly, polysaccharide from *A. brasiliensis* (PLS) reduced PV-1 plaque formation by up to 67% immediately after inoculation without virucidal activity and inhibition of viral adsorption in Hep-2 cells, (Faccin et al., 2007). Hot aqueous extracts of *G. neo-japonicum* exhibited broad-spectrum virucidal activity against enteroviruses causing hand, foot, and mouth disease (HFMD). The polysaccharide fractions reduced EV-A71 and CV-A16 titers in human primary oral fibroblast cells (HPOF), and showed virucidal activity against cell-free EV-A71 without cytotoxicity (Ang et al., 2021).

Beyond the viruses described above, mushroom polysaccharides have shown inhibitory effects on a range of other viruses, spanning animal and plant hosts. *L. edodes* heteroglucan (LNT-I) exhibited strong activity against infectious hematopoietic necrosis virus (IHNV) by direct inactivation, inhibiting replication and modulating immune responses (Ren et al., 2018). In psittacine birds naturally infected with psittacine circovirus (PsCV), oral administration of *β*-(1,3/1,6)-D-glucan from *P. ostreatus* promoted the clearance of viral DNA from blood over several months (Tomasek and Tukac, 2007). The polysaccharide from *H. erinaceus* (HEP) protected the small intestinal mucosal immune barrier in Muscovy ducklings infected with Muscovy duck reovirus (MDRV) (Wu et al., 2018). Moreover, total polysaccharide from *A. auricula* (AAPt) and its sulfated derivatives demonstrated inhibitory effects on Newcastle disease virus (NDV) in chicken embryo fibroblast when administered at pre-, post-, or simultaneously with viral infection, while sulfated derivatives showed superior activity (Nguyen et al., 2012). Furthermore, Lentinan and its sulfated derivative sLNT inhibited tobacco mosaic virus (TMV) infection and proliferation, with sLNT binding to TMV coat protein and activating host defense-related genes (Wang et al., 2013). Overall, mushroom-derived polysaccharides exhibit broad-spectrum antiviral activities against both DNA and RNA viruses through multiple mechanisms. Despite these promising findings, most studies remain confined to *in vitro* or animal models, and systematic clinical validation is still limited (Table 1).

**Table 1:** Antiviral ability of mushroom polysaccharide and its derivatives.

Polysaccharide	Mushroom species	Source	Key structural features	Virus (strain)	Antiviral outcome	Model	Ref
Influenza virus							
Polysaccharide	*C. versicolor*	Fruiting bodies	β-(1 → 3,1 → 4)-glucan, 750 kDa	*Influenza A (H1N1); Avian influenza A (H9N2)*	Reduced weight loss, mortality, viral titers, and pathological lung damage	Mouse; Chicken	Shi et al. (2022)
KS-2	*L. edodes*	Mycelia	α-linked mannose, 60–75 kDa	*Influenza A2 (H2N2)*	Reduced mortality, lung consolidation, and viral titers; prolonged survival	Mouse	Suzuki et al. (1979)
Polysaccharide	*A. luteo-virens*	Fruiting bodies	Not characterized	*Influenza A (H3N2)*	IC50: 7.81 ± 0.12 μg/mL	MDCK cells	Kurskaya et al. (2022)
Herpesvirus (HSV/BoHV)							
Polysaccharide (*β*-glucan)	*A. brasiliensis*	Fruiting bodies	β-glucan	*BoHV-1*	IC50: 140 μg/mL; SI: 9.19	HEp-2 cells	Minari et al. (2011)
Polysaccharide-peptide	*A. brasiliensis*	Fruiting bodies	Polysaccharide–peptide complex	*BoHV-1*	IC50: 160 μg/mL; SI > 12.50	HEp-2 cells	
PLS	*A. brasiliensis*	Fruiting bodies	β-(1 → 6)/α-(1 → 4)-glucan–protein	*HSV-1; BoHV-1*	IC50: 454 μg/mL, %VI: 83.6%, SI > 5.5 (HSV-1); IC50: 634 μg/mL, %VI: 69.2% (BoHV-1)	HEp-2 cells	Yamamoto et al. (2013)
SPLS	*A. brasiliensis*	Fruiting bodies	Sulfated derivative of PLS	*HSV-1; BoHV-1*	IC50: 346 μg/mL, %VI: 82%, SI > 7.2 (HSV-1); IC50: 830 μg/mL, %VI: 53.6% (BoHV-1)	HEp-2 cells	
F3	*A. brasiliensis*	Fruiting bodies	Polysaccharide fraction	*HSV-1; BoHV-1*	IC50: 674 μg/mL, %VI: 77.4%, SI > 3.7 (BoHV-1)	HEp-2 cells	
FR	*A. brasiliensis*	Fruiting bodies	β-(1 → 6)/(1 → 3)-D-glucan	*HSV-1 KOS; HSV-2 str. 333*	Inactive	Vero; GMK-AH1 cells	Cardozo et al. (2014)
FR-S	*A. brasiliensis*	Fruiting bodies	Sulfated derivative of FR	*HSV-1 KOS; HSV-2 str. 333*	SI > 393; EC50: 0.32–8.39 μg/mL (HSV-1); EC50: 0.10–2.86 μg/mL (HSV-2)	Vero; GMK-AH1 cells	
MI	*A. brasiliensis*	Mycelia	β-1,2-D-glucomannan	*HSV-1 [KOS, 29R]; HSV-2 str. 333*	Inactive	Vero cells; Mouse	Cardozo et al. (2013) and Cardozo et al. (2011)
MI-S	*A. brasiliensis*	Mycelia	Sulfated derivative, 86 kDa	*HSV-1 [KOS, 29R]; HSV-2 str. 333*	SI > 439/208/562; EC50: 2.35–17.27 μg/mL; reduced viral titers *in vivo*	Vero cells; Mouse	
TM8	*P. tuber-regium*	Sclerotia	β-(1 → 3)-D-glucan, 57.6–774 kDa	*HSV-1; HSV-2*	Inactive	MDCK cells	Zhang et al. (2003) and Zhang et al. (2004)
TM8-S	*P. tuber-regium*	Sclerotia	Sulfated derivative, DS: 1.14–1.74	*HSV-1; HSV-2*	IC50: 0.6–2.5 μg/mL (HSV-1); IC50: 0.125–1.25 μg/mL (HSV-2)	MDCK cells	
LeP	*L. edodes*	Fruiting bodies	β-(1 → 6)/α-(1 → 4)-glucan	*BoHV-1*	IC50: 0.1 mg/mL; SI > 39.21	HEp-2 cells	Rincao et al. (2012)
HIV							
J, JH	*A. blazei*	Fruiting bodies	55.1/50.6 kDa	*HIV*	IC50 > 50 μM	TZM-bl cells	Zhao et al. (2021)
JS, JHS	*A. blazei*	Fruiting bodies	Sulfated, 30.2–59.5 kDa; DS: 0.35–1.29	*HIV*	IC50: 0.017–0.193 μM	TZM-bl cells	
LA	*P. abalonus*	Fruiting bodies	(1 → 6)-D-glucan, 120 kDa	*HIV*	IC50: 8.7 × 10^−2^ μM for HIV-1 reverse transcriptase	Cell-free enzyme assay	Wang et al. (2011)
G1	*F. punctata*	Fruiting bodies	~151 kDa; Ara: Fuc: Gal: Glc = 1.6:3.8:19.7:19.7	*HIV*	Inhibited HIV-1 protease (19.6% at 50 μg/mL) and integrase activity	Cell-free enzyme assay	Liu et al. (2017)
PSP	*C. versicolor*	Mycelia	~100 kDa	*HIV*	IC50: 150 μg/mL (gp120–CD4 binding); IC50: 6.25 μg/mL (reverse transcriptase)	Cell-free enzyme assay	Collins and Ng (1997)
Hepatitis B virus (HBV)							
LEP-1, LEP-2	*L. edodes*	Fruiting bodies	39.8 kDa; 215.3 kDa	*HBV*	HBsAg inhibition: 27.6, 33.4%; HBeAg inhibition: 26.3, 31.9%	HepG2.2.15 cells	Zhao et al. (2018)
PLWM	*L. edodes*	Waste material	Not characterized	*HBV*	IC50: 24.6–26.1 μg/mL (HBsAg); IC50: 86.2 μg/mL (HBeAg); SI: 8.05–8.10	HepAD38; HepG2.2.15 cells	Jiao et al. (2018)
FVP1	*F. velutipes*	Fruiting bodies	54.78 kDa; Man: Glc: Gal = 1:9.1:2.1	*HBV*	Inhibited HBsAg, HBeAg expression and HBV DNA replication	HepG2.2.15 cells	Zhang et al. (2018)
Sizofiran	*S. commune*	Culture medium	~450 kDa	*HBV*	Increased IFN-γ, anti-HBc, and anti-HBe production; host-mediated mechanism	PBMC cells	Kakumu et al. (1991)
Enteroviruses (EV71, CV-A16, PV-1)							
GFP1	*G. frondosa*	Mycelia	β-(1,6)-D-glucan, 40.5 kDa	*EV71*	Inhibited viral replication and cell apoptosis; blocked VP1 expression	Vero cells	Zhao et al. (2016)
S5	*G. neo-japonicum*	Fruiting bodies	Not characterized	*EV71; CV-A16*	Reduced virus titers; direct virucidal activity	HPOF cells	Ang et al. (2021)
PLS	*A. brasiliensis*	Fruiting bodies	β-glucan complex	*PV-1*	Inhibited viral replication; SI > 9.9	HEp-2 cells	Faccin et al. (2007)
LeP	*L. edodes*	Fruiting bodies	β-(1 → 6)/*α*-(1 → 4)-glucan	*PV-1*	IC50: 0.19 mg/mL; SI > 21.33	HEp-2 cells	Rincao et al. (2012)
Other viruses (NDV, TMV, IHNV, MDRV, PsCV)							
AAP	*A. auricula*	Fruiting bodies	Not characterized	*NDV*	Viral inhibition: 16.78–51.37%	CEF cells	Nguyen et al. (2012)
SAAP	*A. auricula*	Fruiting bodies	Sulfated; DS: 0.22, 1.19, 1.46	*NDV*	Viral inhibition: 24.60–70.9%	CEF cells	
LNT	*L. edodes*	Fruiting bodies	β-(1 → 3)-D-glucan	*TMV*	Inactivation: 83.2%; curative: 58.7%; protection: 71.6%	Tobacco plants	Wang et al. (2013)
sLNT	*L. edodes*	Fruiting bodies	Sulfated LNT; DS: 0.98	*TMV*	Inactivation: 87.4%; curative: 65.0%; protection: 77.2%	Tobacco plants	
LNT-I	*L. edodes*	Mycelia	β-(1 → 3)-glucan, 379 kDa	*IHNV*	Direct inactivation; EC50: 13.20–209.67 μg/mL; immunomodulatory	EPC cells	Ren et al. (2018)
Glucan	*P. ostreatus*	Fruiting bodies	β-(1,3/1,6)-D-glucan	*PsCV*	Reduced PsCV DNA in whole blood	Psittaciformes (in vivo)	Tomasek and Tukac (2007)
HEP	*H. erinaceus*	Fruiting bodies	~16.18 kDa; Glc: Gal: Man: Ara = 22.6:18.8:2.0:1	*MDRV*	Restored injured intestinal mucosal immunity	Muscovy ducklings	Wu et al. (2018)

IC50, Half-maximal inhibitory concentration; EC50, Half-maximal effective concentration; SI, Selectivity index (CC50/IC50 or CC50/EC50); %VI = percentage viral inhibition; DS, Degree of sulfate substitution; IZ, Inhibition zone; NA, Not characterized.

### 3.2 Antibacterial activities of mushroom polysaccharides

Mushroom-derived polysaccharides display broad-spectrum antibacterial activities against Gram-positive, Gram-negative, and drug-resistant bacteria, with varying potency depending on bacterial cell envelope structures, polysaccharide origin, and structural modifications. Generally, Gram-positive bacteria demonstrate higher susceptibility, likely due to the absence of an outer membrane, whereas Gram-negative species require polysaccharides with specific physicochemical properties to overcome their protective lipopolysaccharide layer. Moreover, several mushroom polysaccharides and their derivatives show activity against multidrug-resistant pathogens.

Mushroom polysaccharides exhibit notable activity against Gram-positive bacteria such as *Staphylococcus* sp. (particularly *S. aureus*), and *Bacillus* sp. and *Listeria monocytogenes*, which are widely used as indicator strains in antimicrobial assay due to their clinical and food safety relevance. Polysaccharides isolated from *G. lucidum*, *P. ostreatus*, *L. edodes*, and *A. bisporus* have demonstrated strong inhibitory effects. A polysaccharide fraction (PL2) from *L. edodes* substrate exhibited strong inhibitory effect against *S. aureus* with minimum inhibitory concentrations (MICs) of 25 μg/mL (Zhu et al., 2012). Similarly, exopolysaccharides from *G. lucidum* (EPS) cultured on malt medium inhibited the growth of *B. cereus* with an inhibition zone (IZ) of 23 ± 0.61 mm, outperforming erythromycin (20 ± 0.72 mm) (Mahendran et al., 2013). The favorable efficacy against Gram-positive bacteria may be attributed to their lack of outer membrane, allowing easier access for polysaccharides to disrupt the thick peptidoglycan layer, leading to cell wall destabilization or cytoplasmic leakage. Selective inhibition was reported for exopolysaccharide of *G. applanatum* (EPS), which exhibited a 17.98 ± 0.4 mm inhibition zone and MIC of 1 mg/mL against *S. aureus*, but no effect on *E. coli* (Osińska-Jaroszuk et al., 2014). Chitosan from *G. lucidum* also inhibited the growth of *S. aureus* more effectively than *Pseudomonas aeruginosa* (Savin et al., 2020). Structural modifications further enhance antibacterial potency of mushroom polysaccharides. Sulfated polysaccharides from *P. eryngii* (PEPS) produced a remarkable IZ of 31.8 ± 0.5 mm and MIC < 0.625 mg/mL, far exceeding native PEPS (17.2 ± 0.8 mm, 1.25 mg/mL; Li and Shah, 2014). Notably, *L. monocytogenes*, a facultative intracellular foodborne pathogen, was effectively inhibited by polysaccharides from *P. eryngii* (Li and Shah, 2014) and *G. frondosa* (Zhang et al., 2017) *in vitro*, while PS-F2 from *G. formosanum* (Wang et al., 2011) and Pleuran from *P. ostreatus* (Karácsonyi and Kuniak, 1994) reduced the bacterial loads and improved survival in infected mice. These polysaccharides offer a natural and promising antibacterial strategy for preventing and controlling foodborne bacterial infections.

Gram-negative bacteria often exhibit reduced susceptibility to many natural antimicrobials because of their outer membrane. Nonetheless, several mushroom polysaccharides have shown measurable inhibition against *E. coli*, *P. aeruginosa*, *K. pneumoniae*, *Salmonella* spp. among others. Crude lentinan was found to inhibit five Gram-negative bacteria, with the largest zone (43 mm) against *Acinetobacter* sp. and the smallest (18 mm) against *E. coli* (Sharma et al., 2016). Similarly, mycelia polysaccharides (PAT) from *A. tabescens* showed an inhibition zone of 25.4 ± 0.5 mm against *E. coli* and 18.2 ± 0.9 mm against *Proteus vulgaris*, with MICs of 0. 5 mg/mL and 1 mg/mL, respectively (Zhang et al., 2022). Chitin-glucan complex (ChGC) inhibited growth of *E. coli* and *P. aeruginosa* more effectively than native chitin, achieving IZ of 40 mm and 30 mm, respectively, (Singh and Dutta, 2017). Polysaccharides from *L. edodes* and *G. lucidum* also suppressed *Salmonella* spp., including *S. typhimurium*, *S. enteritidis*, *S. typhi* (Chien et al., 2016; Qin et al., 2018). Although *β*-glucan from *C. versicolor* showed no direct inhibitory effect against *S. typhimurium in vitro*, it significantly enhanced macrophage-mediated bacteria clearance and improved survival in salmonellosis mice, highlighting its potential for host-directed antibacterial therapy (Shi et al., 2016). Beyond human pathogens, mushroom polysaccharides have also demonstrated inhibitory activity against plant-pathogenic Gram-negative bacteria, including *Acidovorax avenae*, *Agrobacterium rhizogenes*, *Agrobacterium tumefaciens*, *Erwinia carotovora*, *Pseudomonas syringae*, *Xanthomonas campestris*, indicating potential applications in plant disease control. Overall, these MIC values are relatively high, suggesting that, despite the promising antibacterial activity, further evaluation of cytotoxicity and the potential for clinical translation is necessary. Moreover, MIC values for the same target organism vary considerably across studies. For instance, MICs for *G. lucidum* polysaccharide fractions against *S. aureus* range from 0.125 mg/mL to over 10 mg/mL. This variability reflects differences in extraction method, purity grade, inoculum density, and assay conditions rather than genuine biological variation, and making aggregate comparisons unreliable. Besides, disk diffusion and broth microdilution assays fail to account for polysaccharide stability under physiological ionic conditions, capacity to penetrate biofilm matrices, or synergy with host immune effectors, all of which are likely to influence *in vivo* outcomes.

Mushroom polysaccharides demonstrate potential in combating multidrug-resistant (MDR) bacteria through both direct and synergistic mechanisms. Sulfated glucans from *G. lucidum* (GS) demonstrated dose-dependent inhibition against a broad range of clinically relevant bacteria, including Methicillin-Susceptible *S. aureus*, *Staphylococcus epidermidis*, *P. aeruginosa*, and *Shigella sonnei*, with inhibition zones of 23 ~ 34 mm at 500 μg/mL. GS further inhibited the growth of antibiotic-resistant *K. pneumoniae*, reducing bacterial survival to 52.8 ± 5.66% after 24 h, and suppressed the acid-fast *Mycobacterium marinum* to 65.0 ± 3.39% survival at 100 μg/mL (Wan-Mohtar et al., 2016). Beyond direct antibacterial effects *in vitro*, *β*-glucans from *L. edodes* effectively reduced bacterial load, inflammatory leukocyte infiltration and protein leakage, thereby alleviating *K. pneumoniae*-induced lung injury (Masterson et al., 2020). AgNP–glucan conjugates from *P. florida* exhibited strong inhibitory activity against multiple antibiotic-resistant *K. pneumoniae* YSI6A. Moreover, the combination of AgNPs-glucan conjugates with conventional antibiotics showed the synergistic antibacterial effect, highlighting their potential as adjunctive therapies for resistant infections (Sen et al., 2013). The synergistic application of mushroom polysaccharides with conventional antibiotics represents a highly viable chemotherapeutic strategy. This approach not only lowers the required dosage of standard antimicrobial drugs, thereby minimizing off-target toxicity, but also effectively resensitizes MDR pathogens to existing treatments, offering a sustainable solution to the antimicrobial resistance crisis. However, except for a limited number of animal infection models, the available antibacterial data against MDR organisms derive almost entirely from *in vitro* assays, and clinical evidence currently is lacking. The molecular basis of the observed MDR inhibition also remains poorly characterized. Whether mushroom polysaccharides interfere with efflux pump expression, alter outer membrane permeability, or suppress resistance gene transfer has not been systematically investigated (Table 2).

**Table 2:** Inhibition ability of mushroom polysaccharide and its derivatives against bacteria.

Polysaccharide	Mushroom species	Characteristics	Microorganism	Effect	Ref
Schizophyllan	*S. commune*	(1 → 3)-β-D-glucan	*M. tuberculosis*	Anti-infecion	Komatsu et al. (1973), Matsumiya and Saito (1977), and Komatsu et al. (1975)
Scleraldehyde		oxidizide schizophyllan	*S. sonnie, B. subtilis, P. aeruginosa, E. coli, V. cholera, S. typhi, P. mirabilus, S. aureus, K. pneumonia, S. dysentriae*	MIC: 3 ~ 4 mg/mL MBC: 6 ~ 8 mg/mL	Jayakumar et al. (2010)
Exopolysaccharide	*G. lucidum*	3,500 ~ 4,500 Da, mainly composed of glucose, ribose, and galactofuranose	*A. avenae*, *A. rhizogenes*, *A. tumefaciens*, *E. carotovora*, *P. syringae*, *X. campestris, E. coli*, *S. aureus*, *Proteus* sp., *B. subtilis*, *P. aeroginosa*, *Klebsiella* sp., *B. cereus*	IZ: 7 ~ 23 mm	Robles-Hernández et al. (2021) and Mahendran et al. (2013)
Polysaccharide	*G. lucidum*	NA	*S. epidermidis*, *S. aureus*, *B. subtilis*, *M. luteus*, *E. coli*, *K. pneumoniae*, *P. aeruginosa*, *P. mirabilis*	MIC: 0.63 ~ 2.5 mg/mL MBC: 2.5 ~ 5 mg/mL	Skalicka-Woźniak et al. (2012)
G	*G. lucidum*	(1,3)-β-D-glucan	*P. aeruginosa, S. enteritidis, S. aureus, S. epidermidis, E. coli, Salmonella* BA54, *L. monocytogenes*, *S. sonnei* Methicillin-Susceptible *S. aureus, K. pneumoniae*, *M. marinum*	No activity	Wan-Mohtar et al. (2016)
GS		sulfated derivatives		IZ: 16 ~ 34 mm MIC: 1 ~ 5 mg/mL MBC: 5 ~ 20 mg/mL	
GpEPS	*G. applanatum*	β-glucan with a-linked glycosyl residues	*S. aureus*, *V. fischer*	IZ: 17.98 ± 0.4 mm IR: 82.6 ± 2.4%	Osińska-Jaroszuk et al. (2014)
PS-F2	*G. formosanum*	heteropolysaccharide, 6–52 kDa	*L. monocytogenes*	Anti-infection, reduce the bacterial loads	Wang et al. (2011)
EP	*P. ostreatus*	NA	*E. coli*	IR: 10% ~ 35%	Wolff et al. (2008)
pleuran	*P. ostreatus*	an alkali-insoluble β-D-glucan	*L. monocytogenes, H. influenzae*	Promote survival of mice	Karácsonyi and Kuniak (1994)
P3a	*P. eous*	5,660 Da, Xyl: Ara: Rib: Rha: Man: Gal: Glu = 2.2: 4.6: 1: 1.5: 1.51: 3.78	*E. coli*, *K. pneumonia*, *S. aureus*, *B. subtilis*	IZ: 8 ~ 15 mm	Gunasekaran et al. (2021)
SP3a		sulfated derivatives, 2,513 Da, Xyl: Rha: Man: Gal: Glu = 4: 1: 3.9: 51.25: 5.14		IZ: 10.4 ~ 14.2 mm	
PEPS	*P. eryngii*	NA	*E. coli, S. aureus, L. monocytogenes*	IZ: 9.8 ~ 17.2 mm MIC: 1.25 ~ 10 mg/mL;	Li and Shah (2014)
Sulphated PEPS		DS: 0.69		IZ: 11.7 ~ 31.8 mm MIC: >0.65 ~ 2.5 mg/mL	
Crude Lentinan	*L. edodes*	NA	*Acinetobacter* sp., *E. coli*, *K. pneumoniae*, *S. typhii*, *V. cholerae*	IZ: 18 ~ 43 mm	Sharma et al. (2016)
Lentinan	*L. edodes*	β-glucan	*K. pneumoniae*	Reduce bacterial count, improve lung function	Masterson et al. (2020)
PL2	*L. edodes*	Glu: Rha: Man = 1: 3.98: 0.94	*E. coli*, *S. aureus*, *S. lutea*	IZ: 7.92 ~ 10.46 mm MIC: 12.5 ~ 100 μg/mL	Zhu et al. (2012)
PAT	*A. tabescens*	29.5 ~ 1,070 kDa, α and β configurations, Man: Rha: Gala: Glu: Ara = 2.87: 6.41: 9.56: 2.53: 0.81	*E. coli*, *P. vulgaris*, *B. subtilis*, *S. aureus*	MIC: 0.5 ~ 4.0 mg/mL IZ: 13.7 ~ 25.4 mm	Zhang et al. (2022)
Crude polysaccharide	*A. auricular-judae*	NA	*E. coli*, *S. aureus*	IZ: 5.55 ± 0.182, 9.84 ± 0.076 mm	Cai et al. (2015)
CVP	*C. versicolor*	750 kDa, β-(1 → 3, 1 → 4)-glucan	*S. typhimurium*	Anti-infection in mice	Shi et al. (2016)
IPS	*G. frondosa*	Rha: Ino: Man = 6.9: 2.1: 1	*S. aureus*, *E. coli*, *B. megaterium*, *L. monocytogenes*	IZ: 13.2 ~ 22.2 mm; MIC: 2.5 ~ 10 mg/mL	Zhang et al. (2017)
IZPS		Rha: Ino: Glu = 4.7: 3.6: 1		IZ: 22.1 ~ 39.7 mm; MIC: <0.625 ~ 2.5 mg/mL	
Melanin-glucan	*F. fomentarius*	NA	*H. pylori*	Inhibition growth	Seniuk et al. (2011)
GLP-NPs	*G. lucidum*	95 ± 7 nm	*S. aureus, B. subtilis, E. coli, Salmonella* sp.	Inhibition growth	Qin et al. (2018)
Chitin	*A. bisporus*	β-1,3-glucans	*S. aureus, P. aeruginosa, B. subtilis, E. coli*	IZ: 20 ~ 30 mm	
Chitin -glucan		β-1,3-glucans	*S. aureus, P. aeruginosa, B. subtilis, E. coli*	IZ: 30 ~ 45 mm	Singh and Dutta (2017)
FVP	*F. velutipes*	NA	*S. aureus, E. coli, B. subtilis*	No activity	Dong et al. (2017)
FVP-Fe		neutralization of FeCl_3_ carbohydrate	*S. aureus, E. coli, B. subtilis*	IZ: 11.87 ~ 15.45 mm	
AgNPs-glucan	*P. florida*	2.445 ± 1.08 nm	*K. pneumoniae*	Synergistic effect	Sen et al. (2013)
Chitin	*L. edodes*	NA	*B. cereus, E. coli, Flavobacterium* sp., *S. typhimurium, V. parahaemolyticus*	IZ: 11.3 ~ 15.1 mm	Chien et al. (2016)
Chitosan		alkaline N-deacetylation, 402.45 ~ 382.73 kDa	*B. cereus, L. monocytogenes, S. aureus, E. coli, Flavobacterium* sp.*, P. aeruginosa, S. typhimurium, V. parahaemolyticus*	IZ: 14.4 ~ 28.8 mm	
Chitosan	*G. lucidum*	Deacetylation: 82.2 ~ 85.1%	*E. coli, S. aureus*	IZ: 16.4 ~ 23.8	Zhu et al. (2018)
Chitosan	*G. lucidum*	47.65 ± 4.63 ~ 65.68 ± 2.43 kDa	*S. aureus, P. aeruginosa*	MIC: 0.625 ~ 2.5 mg/mL	Savin et al. (2020)

IZ, Inhibition zone diameter (mm, disk diffusion); MIC, Minimum inhibitory concentration; MBC, Minimum bactericidal concentration; IR, Inhibition rate (%).

### 3.3 Antifungal activity of mushroom polysaccharides

Invasive fungal infections pose an escalating global health challenge. Mushroom-derived polysaccharides and extracts have been shown to exhibit antifungal effects against several clinically relevant pathogens, though this area remains less extensively studied than their antibacterial and antiviral activities (Mizuno et al., 1995). *β*-glucan isolated from *L. edodes* inhibited the sporulation of *Aspergillus niger*, with MIC of 2.5 mg/mL and a minimum fungicidal concentration (MFC) of 3 mg/mL (Yehia, 2022). The melanin–glucan complex (MGC) and the chitin–glucan–melanin complex (ChGMC) demonstrated strong antifungal activity, with MGC completely inhibiting *C. albicans* growth *in vitro* (Seniuk et al., 2011). Moreover, extracts from *G. lucidum* have shown broad-spectrum antifungal activity against several important fungi, including *Aspergillus* spp., *Fusarium* spp., and *Penicillium* spp., with an MIC as low as 150 μg/mL for *A. niger* (Baig et al., 2015). The extract of *P. ostreatus* inhibited *C. albicans* growth by over 50%, while polysaccharides isolated from the fresh mycelium showed a lower inhibition rate of approximately 40.0% (Wolff et al., 2008). Aqueous extracts from *F. velutipes*, rich in branched carbohydrates containing mannose residues, interfere with the adhesion of pathogenic fungi to host epithelial cells in a dose-dependent manner (Kashina et al., 2016). However, purified polysaccharides from *F. velutipes* (FVP) and their iron complexes (FVP-Fe) exhibited limited direct antifungal effects against yeast, *Rhizopus* and *Aspergillus* species, only producing inhibition zones of 7.94 ± 0.10 mm to 8.00 ± 0.04 mm (Dong et al., 2017). These results highlight the potential of mushroom extracts as eco-friendly biological control agents against both clinical and plant fungal pathogens. Nevertheless, further studies are required to elucidate the specific contribution of purified polysaccharide fractions to antifungal activities. Current evidence suggests that mushroom polysaccharides exert antifungal effects through disruption of fungal cell wall integrity, inhibition of ergosterol biosynthesis, and host-mediated immunostimulation (Karunarathna et al., 2025). Antifungal MIC values for polysaccharide-containing preparations are typically in the range of 0.5–5 mg/mL, which is considerably higher than those of clinical antifungal agents like fluconazole. This raises questions about therapeutic relevance at concentrations achievable *in vivo*. Notably, no studies to date have evaluated mushroom polysaccharides against *C. auris* or azole-resistant *A. fumigatus*, the pathogens representing the most critical unmet needs in antifungal therapy.

Collectively, current evidence demonstrates that mushroom-derived polysaccharides exhibit broad-spectrum antimicrobial activities, although their potency varies depending on the pathogen type, structure and physicochemical characteristics of polysaccharide. Differences in extraction methods, chemical modifications and the formations of nano- or composite system also contribute substantially to these variations. The observed diversity highlights the need for deeper investigation into structure–activity relationships, pathogen-specific mechanisms, and potential synergistic interactions with other antimicrobial agents.

## 4 Mechanism of mushroom polysaccharides against pathogenic microorganisms

Mushroom polysaccharides combat pathogenic microorganisms through two complementary modes of action: modulation of host immunity and direct interference with microbial viability. Immunomodulation enhances both innate and adaptive immune responses, reinforcing host defenses against pathogens. Direct effects involve interfering with microbial adhesion and entry, inhibiting replication, and disrupting microbial cell integrity. Together, these dual strategies provide the basis for the broad-spectrum antimicrobial potential of mushroom-derived polysaccharides.

### 4.1 Immunomodulation of host defense systems

**Figure 2:**
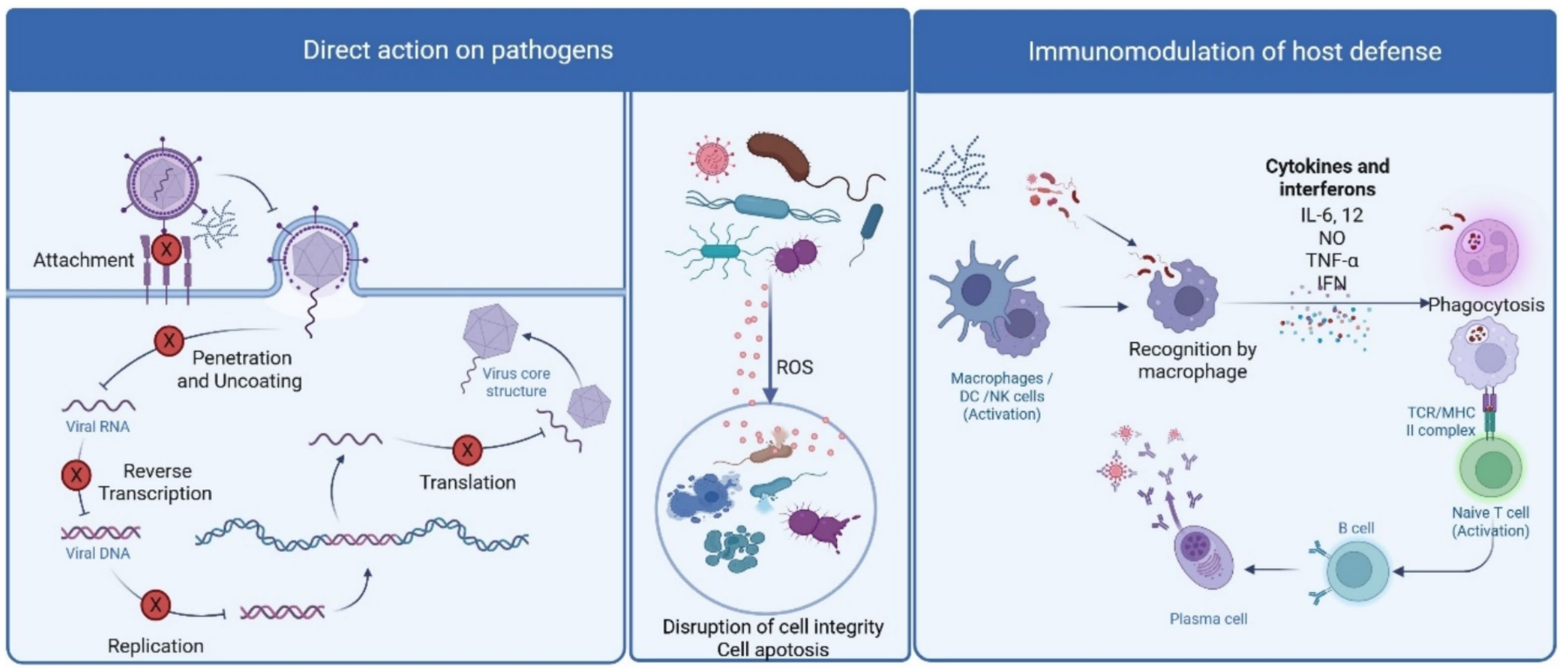
Antimicrobial mechanisms of mushroom-derived polysaccharides. Mushroom polysaccharides exert antimicrobial effects through direct pathogen inhibition and immunomodulation. Direct actions include interference with viral attachment, replication, and microbial cell integrity. Immunomodulatory effects involve activation of macrophages, dendritic cells, and NK cells, enhanced phagocytosis, cytokine and interferon production, and stimulation of adaptive immune responses through T- and B-cell activation. Created with BioRender.com. A publication license has been obtained (Agreement number: SA29SUX9OB), which permits publication under the Creative Commons Attribution License (CC-BY).

A major mechanism of mushroom polysaccharides against pathogens is their capacity to regulate host immune responses. By interacting with pattern-recognition receptors (PRRs) on immune cells (e.g., macrophages, dendritic cells, and NK cells), they trigger cytokine secretion and modulate innate and adaptive responses. This activation promotes pathogen recognition, phagocytosis and clearance, thereby contributing to host protection (Figure 2).

Activation of innate immunity is one of the most well-established functions of mushroom polysaccharides. *β*-glucans stimulate macrophages, dendritic cells, and NK cells, resulting in enhanced phagocytosis and the secretion of effector molecules such as nitric oxide, TNF-*α*, and interleukins (IL). Schizophyllan improved phagocytic and hydrolytic enzyme activities in intraperitoneal macrophages, prolonging survival of *M. tuberculosis*-infected mice (Matsumiya and Saito, 1977). Extracellular polysaccharides from *G. formosanum* stimulated macrophages to trigger an initial inflammatory response, generate NO, and enhance phagocytosis, ultimately protecting mice against *L. monocytogenes* infection (Wang et al., 2011). Similarly, *G. applanatum* EPS induced robust production of IL-6 and TNF-*α* in THP-1-derived macrophages and human PBMCs, contributing to its antibacterial activity (Osińska-Jaroszuk et al., 2014). The polysaccharide fraction FVP1 significantly enhanced the secretion of NO, TNF-α, IL-6, and IL-12 in macrophages, confirming its role in innate immune activation (Zhang et al., 2018). In rodent pneumonia models, *β*-glucans from Shiitake amplified the inflammatory response to *K. pneumoniae* infection by reducing the levels of IL-10 and moderately increasing TNF-α levels, thereby mitigating the lung injury (Masterson et al., 2020). Additionally, β-glucans boosted the immune response to combat influenza virus in mouse and chick models (Shi et al., 2022). They enhanced the expression of dendritic cell-associated C-type lectin-1 (Dectin-1), costimulatory molecules (CD80/86) and cytokines (IL-6, IL-1β, IFN-β) in murine bone marrow dendritic cells, improving antigen presentation during early responses to influenza infection.

Modulation of adaptive immunity further amplifies host defense by promoting antigen-specific and long-lasting protection. Sizofiran regulated both cellular and humoral immune responses in patients with chronic hepatitis B, augmenting T- and B-cell responses specific for viral nucleocapsid antigens (Kakumu et al., 1991). The purified lentinan LNT-I downregulated pro-inflammatory cytokines (TNF-*α*, IL-2, and IL-11) and upregulated antiviral cytokines IFN-1 and IFN-*γ* following IHNV infection, indicating its role in modulating the innate immune response and specific immunity (Ren et al., 2018). Additionally, *H. erinaceus* polysaccharides improved intestinal mucosal immunity in ducklings infected with MDRV, significantly increasing the secretion of secretory immunoglobulin A (sIgA), IFN-γ, and IL-4 (Wu et al., 2018). Together, these findings suggest that mushroom polysaccharides enhance host defense by integrating activation of immune cells, regulation of cytokines and reinforcement of effector molecules, ultimately strengthening infection control. While the immunostimulatory properties of mushroom polysaccharides are well supported by experimental data, several caveats apply to the interpretation of this evidence. The engagement of Dectin-1 and TLR2/TLR4 by *β*-glucans is not receptor-exclusive. Structurally diverse polysaccharides from non-mushroom sources activate overlapping pattern-recognition pathways. It is difficult to attribute observed immune activation specifically to mushroom-derived β-glucans without appropriate structural controls. In addition, at high concentrations, excessive innate immune activation is a concern that has not been systematically evaluated, particularly in the immunocompromised patient populations.

### 4.2 Direct actions on pathogens

Beyond immune modulation, mushroom polysaccharides also exert direct antimicrobial effects by disrupting critical steps in microbial infection, including interference with pathogen attachment and entry, inhibition of intracellular DNA replication. The polysaccharide-peptide PLS and *β*-glucan from *A. brasiliensis* inhibited BoHV-1 replication by interfering with the early events of viral penetration (Minari et al., 2011). LNT blocked TMV infection by binding either to viral particles or host receptors, thereby preventing viral adsorption and subsequent entry into host cells, while simultaneously increasing the expression of defense-related genes in plant tissues to enhance antiviral effects (Wang et al., 2013). Sulfated polysaccharides exert their antiviral effects by competitively binding to viral particles via electrostatic interactions between negatively charged sulfate groups and positively charged glycoprotein on the viral surface. FR-S and MI-S from *A. brasiliensis* significantly suppressed HSV attachment, adsorption, penetration, and cell-to-cell spread (Cardozo et al., 2014; Cardozo et al., 2011). Similarly, S-TM8, enriched with the negative charges presenting on the polymer chain, effectively prevented HSV attachment and invasion through strong interactions with glycoproteins on the viral surface (Zhang et al., 2004). These findings suggest that interference with viral entry is highly dependent on structural modifications, particularly sulfation, which enhances polysaccharide affinity for viral proteins.

Mushroom polysaccharides also inhibit key enzymes essential for microbial metabolism and replication. The polysaccharide from *A. brasiliensis* and its sulfated derivatives inhibited herpesvirus replication, interfering with viral penetration, uncoating and protein synthesis (Yamamoto et al., 2013). *L. edodes* polysaccharides significantly suppressed the secretion of HBsAg and HbeAg and strengthened the inhibition effect of Lamivudine on HBV DNA replications (Jiao et al., 2018). *G. frondosa* polysaccharide inhibited EV71 replication by suppressing viral VP1 protein expression and genomic RNA synthesis (Zhao et al., 2016). Furthermore, polysaccharide fraction from *F. punctata* inhibited HIV-1 protease and integrase (Liu et al., 2017), while abalone mushroom polysaccharides blocked HIV-1 reverse transcriptase (Wang et al., 2011). These results indicate that mushroom polysaccharides may act as natural enzyme inhibitors, targeting critical steps in viral replication.

In addition, several mushroom polysaccharides exhibit direct bactericidal and virucidal effects. Polysaccharides from *A. tabescens* (PAT) inhibited the growth of *E. coli* by targeting the microbial cell membrane and disrupting its integrity, leading to the leakage of intracellular contents (Zhang et al., 2022). PAT also induced microbial cell apoptosis by increasing reactive oxygen species (ROS) generation and impairing DNA amplification. Similarly, the strong antibacterial activity of AgNPs-glucan conjugates against *Klebsiella pneumoniae* YSI6A was possibly attributed to ROS-induced damage to cellular macromolecules, which was suppressed in the presence of the ROS scavenger histidine (Sen et al., 2013). Moreover, LNT-I exhibited direct virucidal activity against IHNV. accompanied by suppression of viral enzyme activities (Ren et al., 2018).

In summary, mushroom polysaccharides exert antimicrobial protection through integrated immunomodulatory effects and direct interference with microbial viability. These two modes of action are not equivalent across different polysaccharide types or pathogen categories. Many mushroom polysaccharides, particularly high MW *β*-glucans, primarily exert host-mediated effects rather than direct antimicrobial action. *In vitro* MIC values are poor predictors of *in vivo* efficacy, which is heavily influenced by the immunological competence of the host. In contrast, chemically modified derivatives, such as highly sulfated polysaccharides and AgNP conjugates, exhibit stronger direct antimicrobial activity. However, their cytotoxicity at effective concentrations warrants careful evaluation. This mechanistic distinction has significant implications for therapeutic development in that immunomodulatory polysaccharides are best suited as adjunctive agents for immunocompromised patients, while directly acting derivatives may be more appropriate for topical applications or in combination with conventional antimicrobials.

## 5 Clinical translation challenge and future perspectives

Among the polysaccharide preparations, only a small number have achieved regulatory approval and sustained clinical use, providing the most direct evidence of therapeutic potential in human populations. *P. umbellatus* polysaccharide (PUPS) was approved by China’s State Food and Drug Administration in 1990 in both capsule and injectable forms for treatment of chronic hepatitis B encompassing 11,703 reported cases, which demonstrated consistent therapeutic efficacy (Guo et al., 2019). The combination treatment with PUPS and hepatitis B vaccine, interferon, acyclovir, or iRNA increased viral clearance and immune recovery, outperforming monotherapies. These findings supported a role for PUPS as an immunomodulatory adjunct that amplifies antiviral host responses rather than acting as a direct antiviral. Lentinan has been approved in Japan and China as an immunoadjuvant for gastric and other cancers since the 1980s. A review of 9,474 cancer cases from 135 studies confirmed benefit in improving quality of life and chemotherapy response rates (Zhang et al., 2019). Early Phase I/II placebo-controlled trials of lentinan in 98 HIV-positive patients provided initial evidence of immunostimulatory activity in an infectious disease context, though without demonstrating direct antiviral efficacy (Gordon et al., 1998). Beyond these preparations, no randomized controlled trials have been conducted for bacterial or fungal infection indications, and the existing HBV clinical data largely predate current trial methodology standards, limiting direct comparability with contemporary direct-acting antiviral therapies.

The pharmacokinetic profile of lentinan illustrates the delivery constraints common to this class of compounds. Following intravenous administration, lentinan exhibited biphasic plasma elimination with preferential hepatic Kupffer cell distribution and lysosomal metabolic degradation (Mu et al., 2026). Oral bioavailability was severely restricted by the large hydrodynamic radius and gastrointestinal enzymatic degradation of high MW polysaccharide chains (Xu et al., 2026). These constraints explain why approved clinical formulations have required parenteral administration, substantially limiting patient acceptability and scalability. Nanoencapsulation offers a promising route to improving oral delivery. *P. ostreatus* polysaccharides encapsulated in chitosan-alginate nanoparticles resulted in a 65% improvement in bioavailability (Ma et al., 2025). Cryo-milled *β*-glucan nanoparticles achieved sustained release and enhanced epithelial permeation compared with free polysaccharide solutions (Chen et al., 2024). Whether these gains translate to clinically meaningful systemic exposure in humans remains to be established. The broader therapeutic application, long-term stability, and regulatory acceptability of nanoformulation approaches require systematic evaluation before clinical development.

Recent reviews have emphasized that filling these pharmacokinetic, safety, and dose–response data gaps is a prerequisite for meaningful clinical translation of polysaccharides (Xu et al., 2026). Although polysaccharides are traditionally regarded as safe compounds for their dietary history, industrially purified and chemically modified preparations require systematic toxicological evaluation. Chronic toxicity, immunoallergic potential, and hepatorenal burden of concentrated polysaccharide preparations have not been adequately characterized, and the cytotoxicity of highly sulfated derivatives remains poorly defined. In addition, the process optimization to ensure batch-to-batch structural consistency, including reproducible sulfation levels and molecular weight distributions, is required. Clinical efficacy needs to be established through randomized controlled trials in defined infectious disease indications, rather than inferred from *in vitro* data. Pharmacokinetic characterization covering absorption, distribution, metabolism, and excretion of specific candidate preparations in humans is equally essential. Scalable production strategies such as biotransformation and nanoencapsulation should also be prioritized to maintain structural consistency across batches and achieve adequate bioavailability.

Future progress in this field can be greatly advanced by several emerging developments. Advances in glycomics, size-exclusion chromatography with multi-angle light scattering (SEC-MALS) and solution-state NMR are making multi-parameter structural characterization increasingly feasible. The growing availability of patient-derived organoids and humanized mouse infection models removes a long-standing barrier to physiologically representative mechanistic studies. Combinatorial approaches pairing polysaccharide immunostimulants with conventional antimicrobials represent an underexplored strategy that could extend therapeutic utility beyond current frameworks. In addition to their potential in systemic therapeutics, mushroom polysaccharides offer promising applications in food preservation and sustainable agriculture, where their activity against spoilage organisms and plant pathogens has been documented. Importantly, their favorable safety profiles and renewable sourcing make them attractive as candidates for therapeutic application in infection control. Overall, mushroom polysaccharides represent a structurally diverse and pharmacologically versatile resource whose full antimicrobial potential remains to be realized.
